# Editorials

**Published:** 1911-04

**Authors:** 


### EDITORIALS

The Business Office of the *Journal-Record* is 1 1-2 to 5 1-2 South Broad Street.
The Editorial Office is 1014-15 Century Building.
Address all Business Communications to *Journal-Record of Medicine*, 1 1-2 to 5 1-2 South Broad Street.
Make remittances payable *to The Journal-Record of Medicine.*
On matters pertaining to the Editorial and Original Communications, address Edgar G. Ballenger, M. D., Atlanta, Ga.
Reprints of Original Articles will be furnished at cost price. Requests for the same should always be made in THE MANUSCRIPT.
We will present, postpaid, on request, to each contributor of an original article, twenty (20) marked copies of *The Journal-Record of Medicine* containing such article.

### THE ROME MEETING OF THE MEDICAL ASSOCIATION OF GEORGIA.

The annual meeting of the Medical Association of Georgia was held in Rome, April 19, 20 and 21, under very auspicious conditions and with good attendance. The papers were rather better than usual, and the discussions were snappy and to the point. Dr. E. C. Davis made a splendid president, holding the men to their allotted time, and showing familiarity with the workings of the Association, both of which are essential qualities for the president to possess.

Practically nothing happened to mar the scientific proceeding until the afternoon of the second day, when it was announced that an error had been made in making out the program and that the election of officers would not be held as stated in the program on Thursday afternoon, but on Friday. After much discussion it was decided that the meeting should not be held until the last day. This vexing and annual subject seems to be a difficult problem to solve. Some think the election should be held on the day in which the largest crowd is present, who could thus have "a say" in electing the officers; others think the crowd on the third day would be much too small if this interesting event be held on the second day, and so, from year to year, we discuss the advantages and disadvantages of the election day, without coming any nearer to a satisfactory solution.

The president made many valuable suggestions in his address, and we hope in the near future to see them carried out.

The Association decided to have a State Medical Journal, hereafter, instead of the transactions, which, undoubtedly, have for years outlasted their usefulness. The affairs of the Association should be decidedly advanced by a good monthly journal, but after the details of the plans described by Editor McCormack, of Kentuck, we cannot feel wholly in accord with the advisability of following *all* of the suggestions made. For instance, is it entirely right for the members of the Association to decline to buy, or threaten to decline to buy, the wares of drug, instrument and book concerns, because said houses do not find it profitable to advertise in the official organ? Such plans were recommended and have been used by other state journals before, but again, we ask, is it giving the business houses a square deal? Are the finances of the Association such that we must beg—or force—advertisers to contribute to our funds, regardless of the service rendered by our advertising pages? Such a boycott might be worthy of the bricklayers' union, but is it becoming to the dignity of the medical profession? Would not the majority of us feel humiliated to have our Association father such a petty scheme to extort money from unwilling advertisers to defary our expenses?

Thus would we pauperize the Medical Association of Georgia.

The following officers were elected:

President, Dr. M. L. Fitch, Carrollton; first vice-president, Dr. R. M. Harbin, Rome; second vice-president, Dr. T. R. Bradley, Augusta; councilor, first district, Dr. J. L. Hiers, Savannah; councilor, second district, Dr. J. G. Dean, Dawson; councilor, third district, R. H. Stovall, Vienna; councilor, fourth district, Dr. C. L. Williams, Columbus.

Delegates to American Medical Association, Dr. W. N. Daughy, Augusta, alternate, Dr. E. G. Ballenger, Atlanta; chairman board of councilors, Dr. W. W. Pilcher, Warrenton; secretary and editor Medical Journal, Dr. W. C. Lyle, Augusta, holds over.

Next meeting place is Augusta, and the time, third Wednesday in April, 1912.

Dr. W. C. Lyle was made editor of the Medical Journal, and his salary increased to $150.

